# Postpartum left circumflex to left atrial fistula: A rare presentation

**DOI:** 10.1016/j.radcr.2022.09.029

**Published:** 2022-09-28

**Authors:** James Hwang, Cameron J. Overfield, Philip A. Araoz

**Affiliations:** a Mayo Clinic Alix School of Medicine, Jacksonville, FL, USA; b Department of Radiology, Mayo Clinic, Jacksonville, FL, USA; c Department of Radiology, Mayo Clinic, 200 1st St SW, Rochester, MN 55902, USA

**Keywords:** Coronary artery fistula, Pregnancy, Heart failure

## Abstract

Coronary artery fistulas (CAFs) are rare and often asymptomatic, but severe complications can occur, resulting in heart failure and cardiac arrhythmia. They have been associated with iatrogenic or traumatic injuries as well as systemic inflammatory conditions. However, there have been very few documented cases of pregnancy associated CAFs. We observed a case of left circumflex to left atrium fistula in a 37-year-old female presenting with insidious onset of progressive dyspnea during pregnancy.

## Introduction

Coronary artery fistula (CAF) is a rare and abnormal connection between coronary arteries and cardiac chambers [1]. Most CAFs are congenital but others can be acquired through iatrogenic or traumatic injuries and systemic inflammatory conditions. Although anatomy of CAFs vary, common origins include the right and left coronary arteries with connections to the right ventricle or right atrium [1].

## Case report

A 37-year-old female presented with exertional dyspnea, heart murmur, and arm paresthesia during pregnancy and was diagnosed with a left circumflex (LCX) to left atrium (LA) fistula on cardiac gated computed tomography angiogram (CTA). This gravida 2 para 2 patient was referred to our center after an insidious onset of exertional dyspnea during 2 pregnancies, the second of which terminated in cesarean section.

**Fig. 1:**
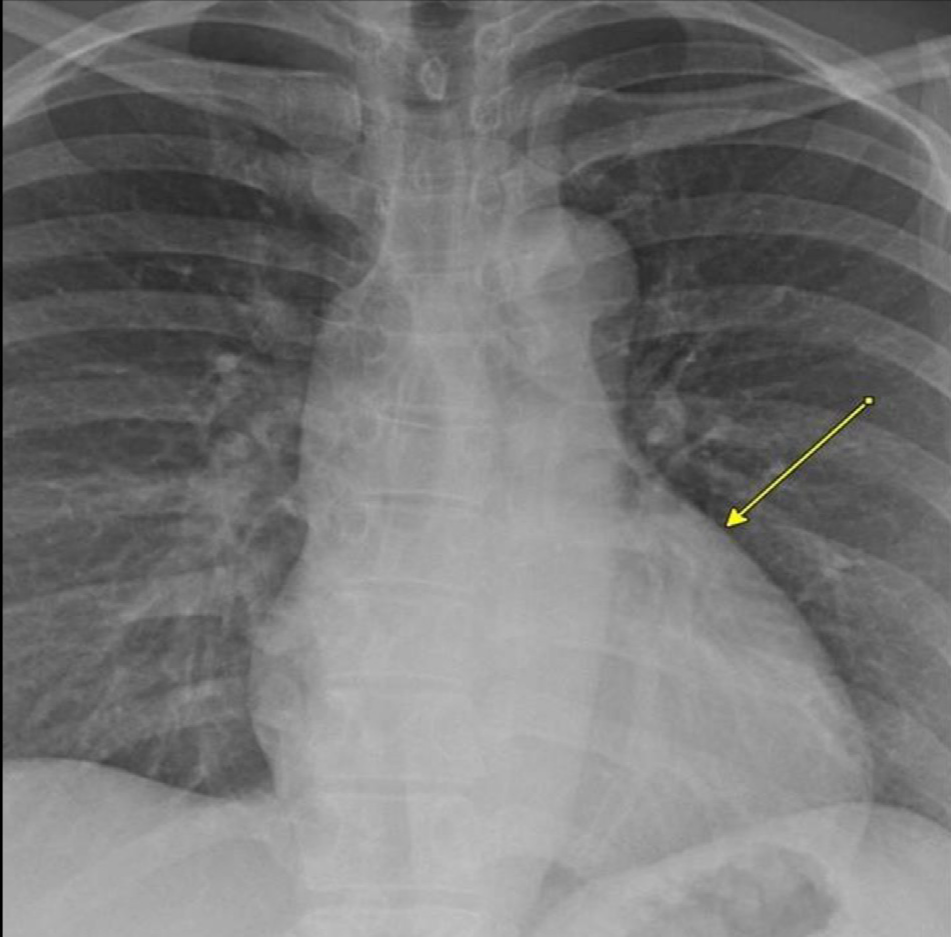
PA chest radiograph: mildly enlarged left atrial sillouhette (yellow arrow).

**Fig. 2:**
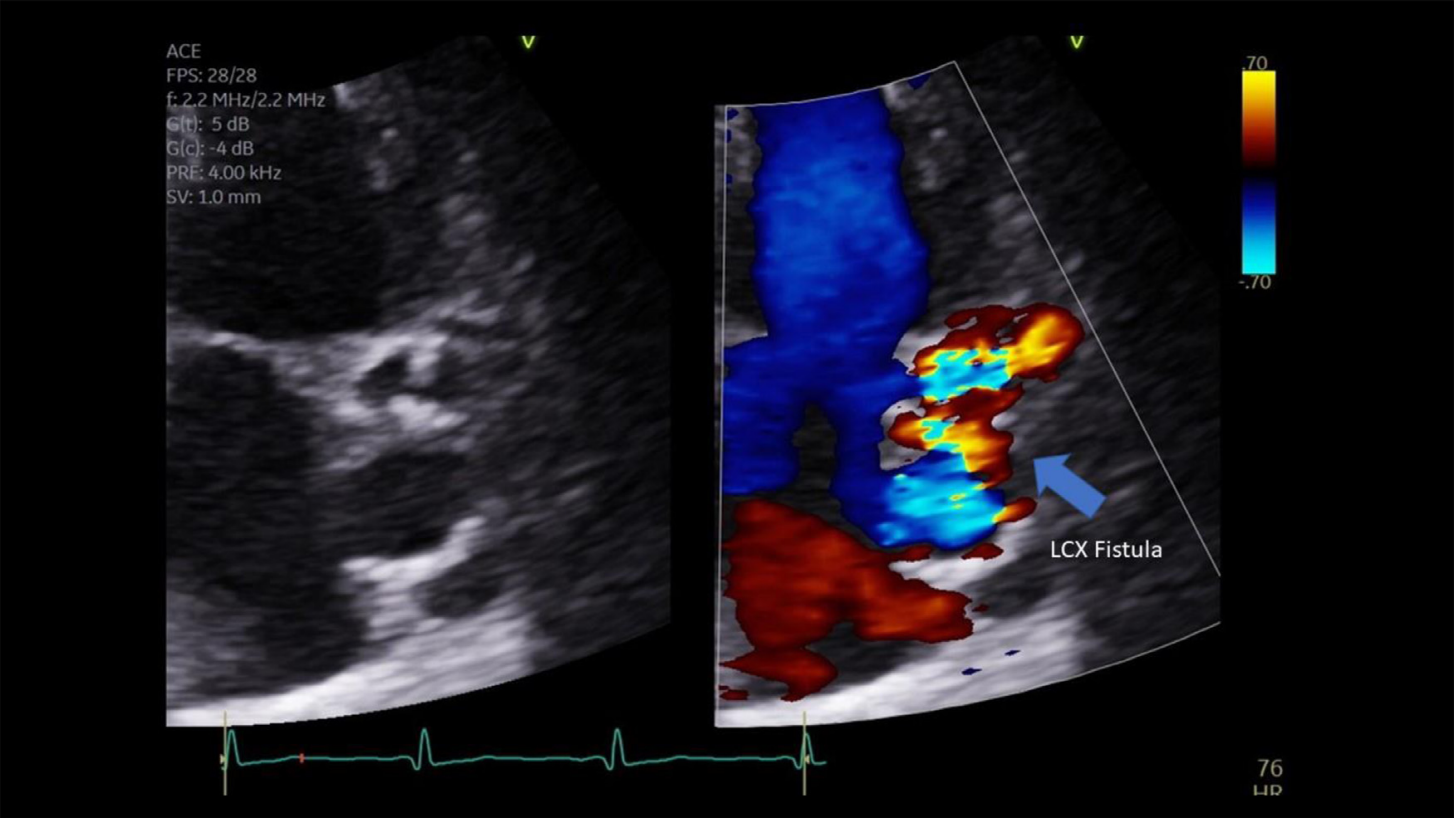
Grayscale and Doppler echocardiogram: the LCX CAF is demonstrated with color aliasing (blue arrow) connecting to the left atrium.

**Fig. 3:**
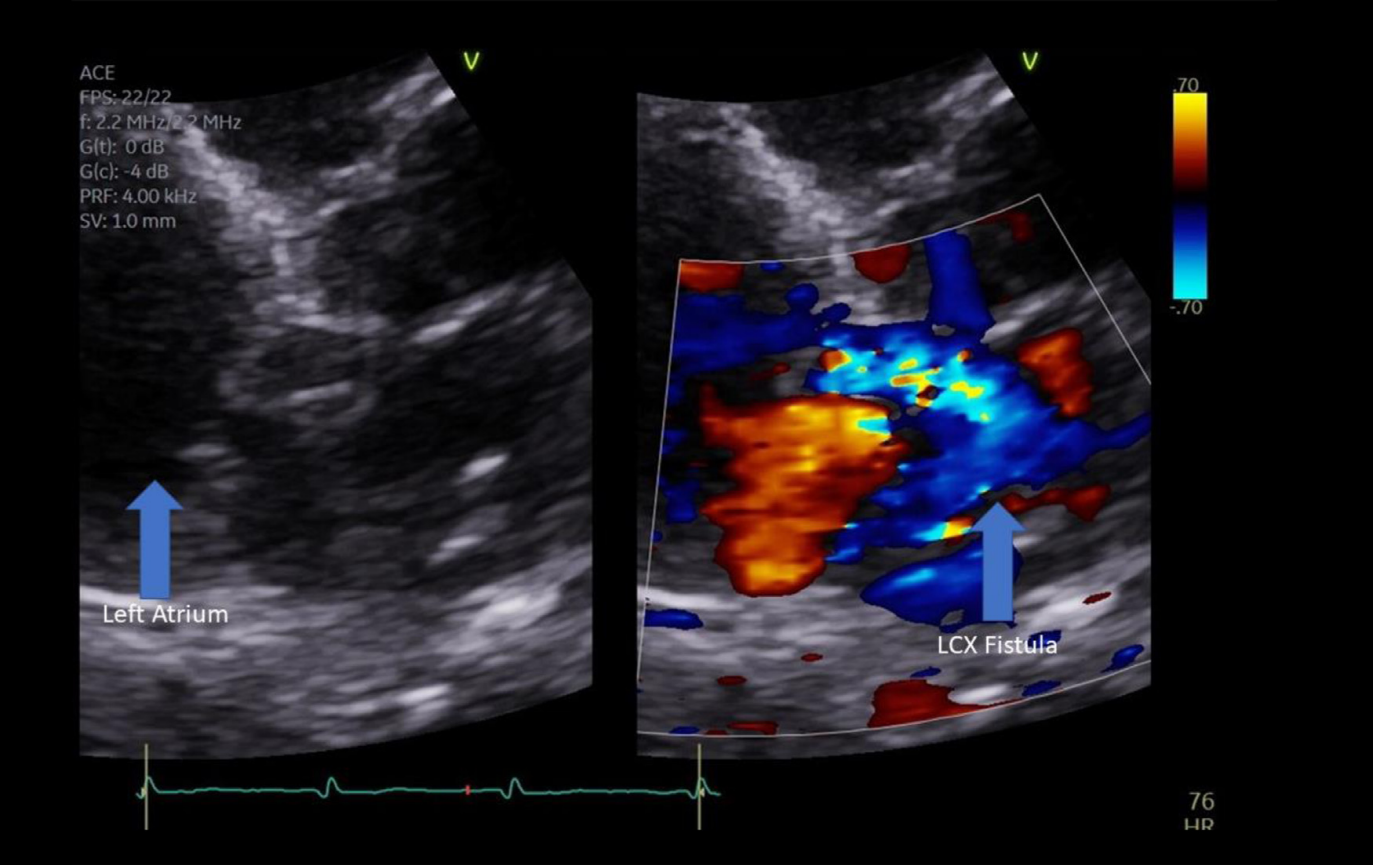
Grayscale and Doppler echocardiogram: turbulent flow from the LCX CAF (blue arrow in the right image) connecting to the left atrium (blue arrow in the left image).

**Fig. 4:**
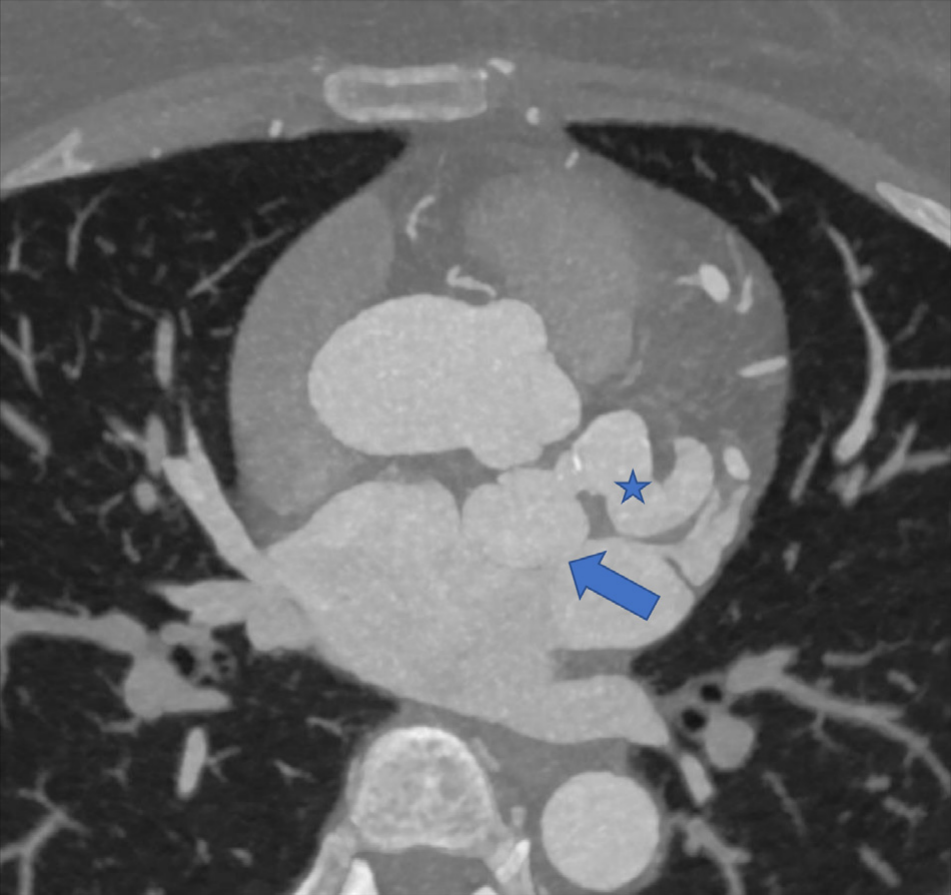
Axial contrast-enhanced CTA maximum intensity projection image: the distal portion of the LCX CAF (blue star) connects to the left atrium (blue arrow) near the left atrial appendage.

**Fig. 5:**
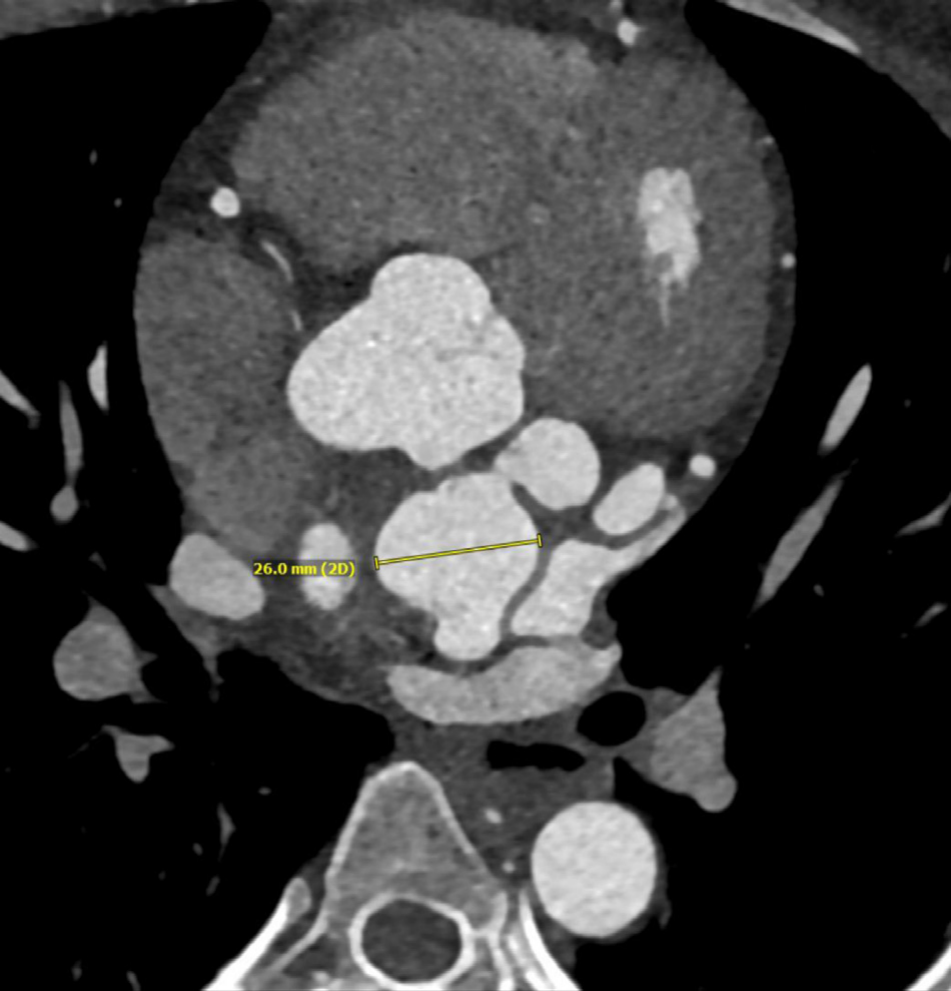
Contrast-enhanced axial CTA image: aneurysmal dilation of the LCX CAF measures up to 26 mm (ruler measurement).

**Fig. 6:**
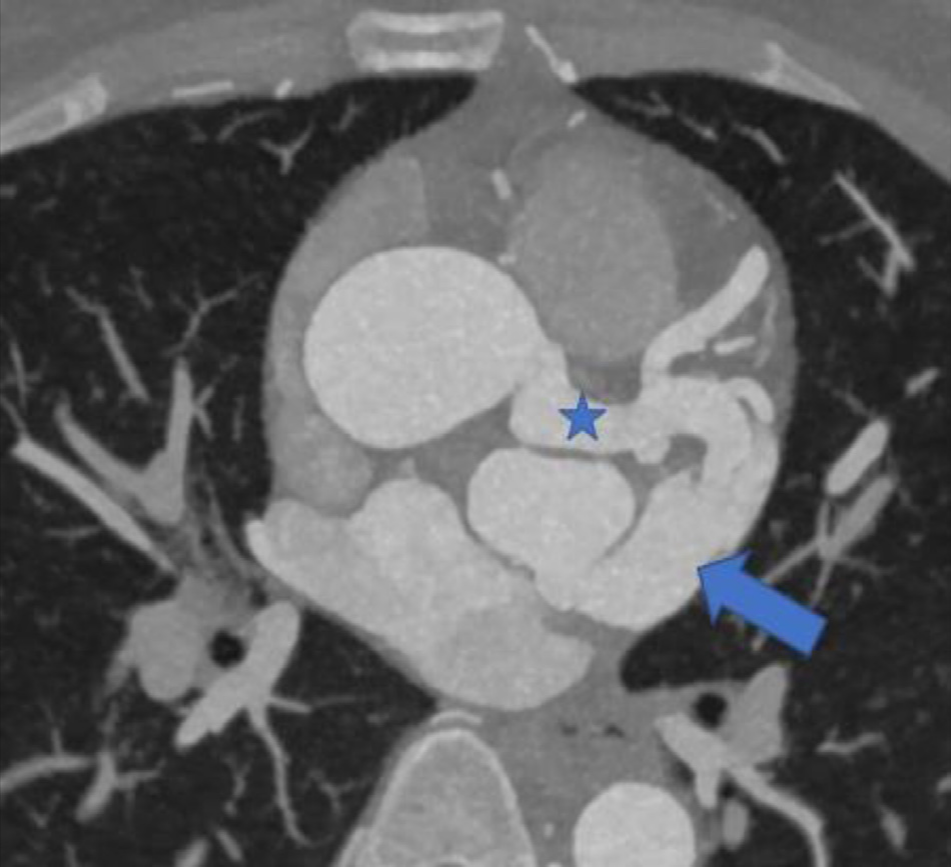
Axial contrast-enhanced CTA maximum intensity projection image: the proximal portion of the LCX CAF (blue arrow) is demonstrated connecting to the dilated left main coronary artery (blue star).

**Fig. 7:**
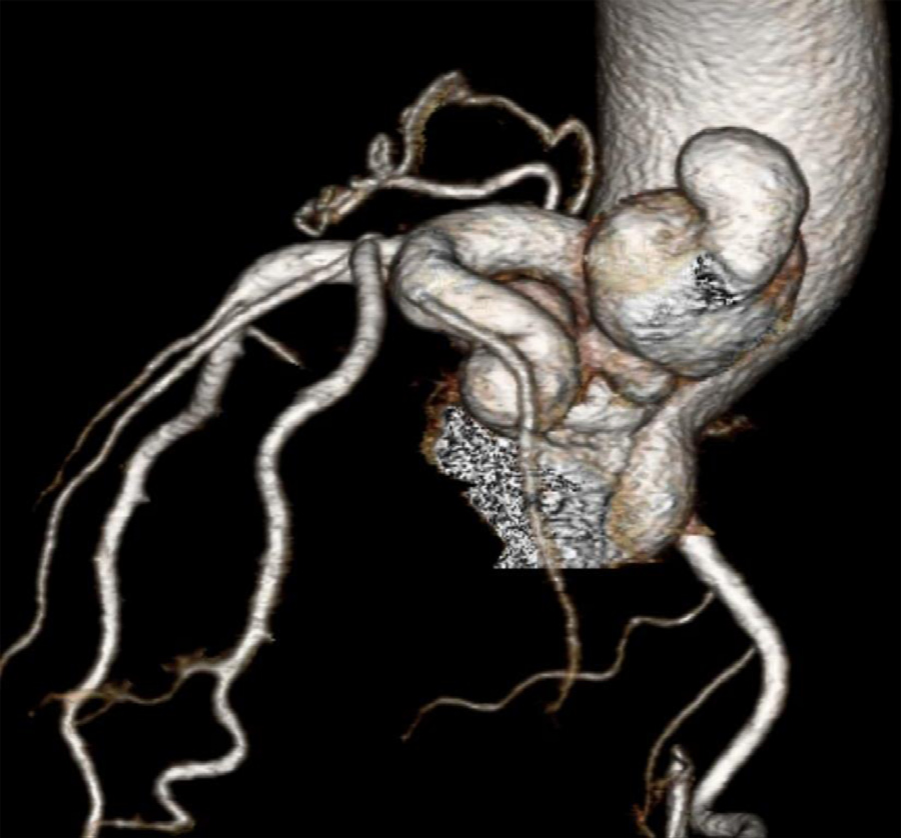
3D reconstruction from cardiac CTA: dilated and aneurysmal LCX CAF.

PA chest radiograph demonstrated a mildly prominent left atrium and tortuous thoracic aorta (Fig. 1). Transthoracic echocardiography revealed LCX to LA CAF (Fig. 2) with turbulent flow into left atrium (Fig. 3), moderately enlarged left atrium, otherwise, the remainder of the cardiac chambers demonstrated normal sonographic characteristics. Cardiac CTA confirmed the tortuous and aneurysmal CAF seen on axial and axial maximum intensity projection images (Figs. 4-Fig. 5, Fig. 6), revealing aneurysmal dilations of the involved coronaries to up to 26 mm (Fig. 7) and several calcified and noncalcified atherosclerotic plaques within the aneurysmal LCX. The left main coronary artery was dilated up to 12 mm in greatest double oblique measurement. The proximal and mid LAD were dilated measuring up to 6 mm in greatest double oblique measurement. The proximal LCX measured up to 10 mm in greatest oblique measurement. Branches of the LCX included a sizeable and mildly tortuous first obtuse marginal (OM) branch and a second smaller OM more distally. After the second OM, the LCX followed a tortuous posterosuperior course before eventually communicating with the left atrium. The fistulous connection was closest to the left atrial appendage. The left atrium was mildly enlarged, otherwise the remainder of the cardiac chambers were normal in size without regional wall motion abnormalities or resting first-pass myocardial perfusion defects.

**Fig. 8:**
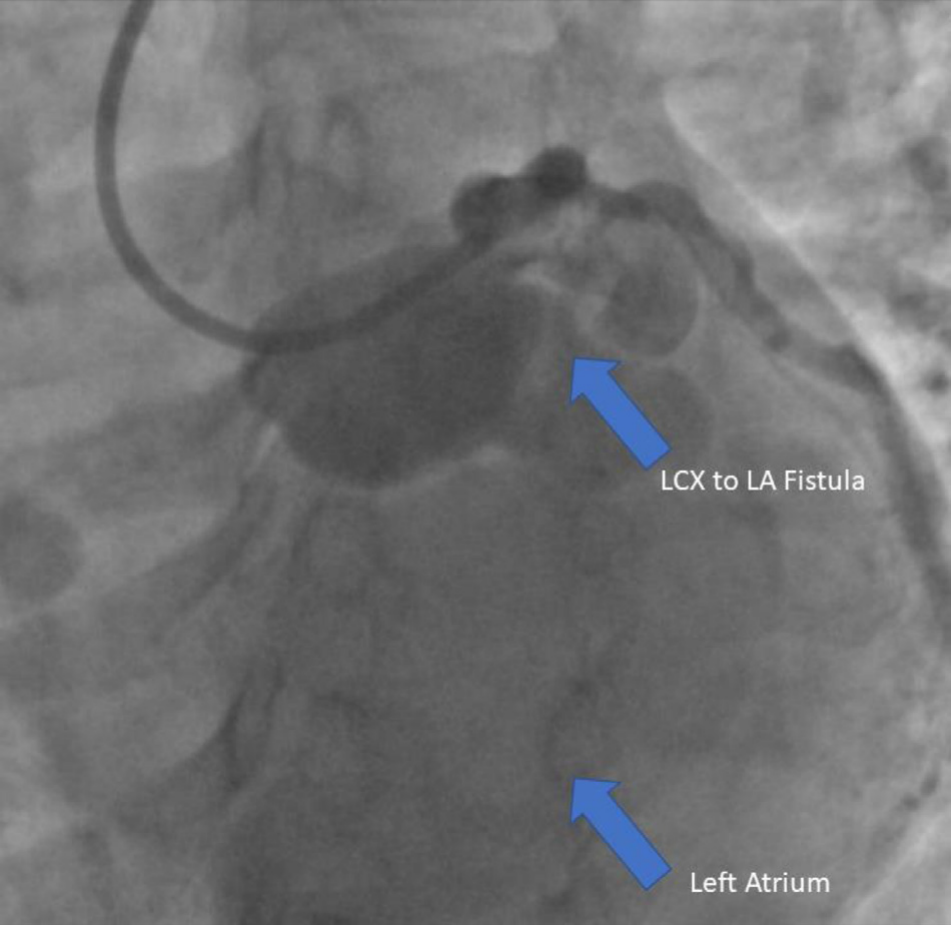
Direct cardiac angiogram: the tortuous and dilated LCX CAF (superior blue arrow) is visualized with subsequent abnormal contrast opacification of the left atrium (inferior blue arrow).

Direct cardiac catheterization of the LCX redemonstrated the LCX to LA CAF (Fig. 8) with contrast opacification of the LA prior to treatment with coil embolization. The patient tolerated the procedure well without complications. Postoperative warfarin and aspirin therapy was prescribed, and the patient was discharged the next day.

## Discussion

Overall, CAFs are a rare phenomenon present in less than 0.002% of the general population. They are rare among cardiac anomalies (0.2%-0.4%) and even coronary anomalies (14%) [1]. A few case reports of pregnancy associated CAFs have been described in the literature [2]. A young, asymptomatic, otherwise healthy, individual raises a clinical dilemma of whether the abnormality should be considered congenital or acquired. Many congenital or acquired CAFs may remain asymptomatic but in the appropriate clinical context severe complications can occur, including those associated with cardiac volume overload, left ventricular hypertrophy, heart failure, and arrhythmia [1]. Enlarged coronary artery branches may produce a coronary steal phenomenon and myocardial ischemia. Endocarditis and hemopericardium are other possible sequelae of CAFs.

An initial diagnostic approach will include physical exam, chest radiographs, and electrocardiogram (ECG). ECG may show the effects of left ventricular volume overload and potentially ischemic changes; however, ECG may be unremarkable [3]. Chest radiographs are generally normal but may show global cardiomegaly or specific chamber enlargement when there is a large, high-flow left-to-right shunt [4].

More advanced diagnostics will include echocardiography, CT angiography, magnetic resonance angiography (MRA), and invasive angiography [5]. Transthoracic or transesophageal echocardiography can demonstrate the fistulous connection, turbulent flow, and functional information about the cardiac chambers without ionizing radiation. Limitations include poor visualization of the distal segments if the vessel is curvilinear along the epicardial surface, limited visualization of smaller collateral vessels, and the inability to detect obstructive coronary artery disease [6]. CTA, MRA, and invasive angiography demonstrate superior anatomic detail and, in the case of CTA, good spatial and temporal resolution [1]. MRA serves as an alternative in children and individuals who need to undergo repeated follow-up imaging [7]. Invasive angiography used to be the reference standard for diagnosis and therapeutic embolization; however, its use in diagnosis has been replaced by non-invasive modalities with better delineation of the complex anatomy of abnormal communications [1].

Treatment depends on size and symptoms. Small and asymptomatic CAFs warrant only observation. Small, symptomatic CAFs can be managed with antiplatelet therapy and antibiotics to prevent endocarditis. Large fistulas are best suited for surgical ligation. Percutaneous transcatheter treatment is indicated if the anatomy is favorable, for example, a single narrow drainage site and a proximal fistula origin [1]. Our patient's symptomatic presentation and favorable anatomy made coil embolization a suitable therapy.

## Patient consent

Written, informed consent for publication of this patient's case was obtained by the patient.
